# Maladaptive cognitive regulation moderates the mediating role of emotion dysregulation on the association between psychosocial factors and non-suicidal self-injury in depression

**DOI:** 10.3389/fpsyt.2023.1279108

**Published:** 2023-11-30

**Authors:** Yuqi Ge, Yang Xiao, Mingzhu Li, Lei Yang, Peihua Song, Xueni Li, Hao Yan

**Affiliations:** ^1^Peking University Sixth Hospital, Peking University Institute of Mental Health, Beijing, China; ^2^NHC Key Laboratory of Mental Health, National Clinical Research Center for Mental Disorders (Peking University Sixth Hospital), Beijing, China

**Keywords:** non-suicidal self-injury (NSSI), depression, emotional dysregulation, psychosocial factors, maladaptive cognitive regulation strategies

## Abstract

**Introduction:**

Non-suicidal self-injury (NSSI) is highly prevalent in depression, and is associated with psychosocial factors, emotion dysregulation, and strategies of cognitive emotion regulation. However, the internal combination and interactions of these risk factors in depression remain unclear.

**Methods:**

Data from 122 patients with depression, including 56 with NSSI and 66 without NSSI, were analyzed. Self-rating scales were used to assess psychosocial factors, emotion dysregulation, and cognitive regulation strategies. Sparse partial least squares discriminant analysis (sPLS-DA) was employed to explore internal combinations in each profile. A moderated mediation model was applied to examine their interactional relationship.

**Results:**

The results identified an NSSI-related psychosocial profile characterized by high neuroticism, childhood trauma, poor family functioning, and low psychological resilience. Emotion dysregulation, including high levels of alexithymia, anhedonia, and emotion regulation difficulties, mediated the association between this psychosocial profile and NSSI. The mediated effect was further moderated by maladaptive cognitive regulation strategies.

**Limitations:**

Lack of sufficient information on NSSI frequency and severity. Relatively small sample size for discussing the impact of gender and age of depressive patients with NSSI.

**Conclusion:**

These findings hold important implications for the prevention, treatment, and rehabilitation of NSSI.

## 1 Introduction

Non-suicidal self-injury (NSSI) refers to the deliberate, direct injuring bodily tissues without suicidal intent and is socially unacceptable (1). NSSI is highly prevalent among adolescents and clinical samples with mental disorders (2). Its prevalence has been increasing, particularly in patients with depression (3). NSSI represents a significant challenge for mental health professionals and has been linked to various adverse outcomes, including increased risk of suicidal behaviors and a considerable impact on overall well-being.

Prior research has suggested that self-harm behaviors may be influenced by a combination of genetic traits, mental disorders, and various psychosocial and familial factors (4, 5). Despite the lack of fully elucidated risk factors and their specific mechanisms, NSSI is commonly motivated by the need to cope with emotional distress and may serve as a strategy for regulating negative emotions (6). Nock proposed an integrated theoretical model of the development and maintenance of NSSI (7). This model identifies two primary categories of factors contributing to the risk of NSSI, distal psychosocial risk factors, including personality traits, childhood trauma, and poor family functioning, lead to intrapersonal and interpersonal vulnerability in emotion dysregulation. A growing body of evidence suggest that emotion dysregulation plays a pivotal role in the association between depression and NSSI (8). Depression, characterized by significant and persistent depressed mood, lack of pleasure and loss of interest, accompanied by cognitive and behavioral changes of varying degrees, is a widely prevalent psychiatric disorder. Beyond its core symptoms, depression is also associated with a complex web of psychosocial factors (9). Previous studies on risk factors for NSSI in depression have mainly focused on psychosocial factors and emotion dysregulation, with some indicating the mediating role of emotion dysregulation. In a study of adolescents, the association between childhood maltreatment and NSSI-related clinical outcomes was partially explained by impulsivity (10). Another study presented the association of poor family functioning and adolescent NSSI, which was mediated by depression (11). There was also a study summarizing that neuroticism positively predicts depression and NSSI behaviors, and affects NSSI through the mediating effect of emotion regulation and depression (12). However, most studies have examined individual risk factors in isolation, limiting our understanding of the internal combinations of psychosocial factors and emotion dysregulation associated with NSSI.

Additionally, the affect regulation hypothesis (13) has been developed to explain the impact of emotion regulation strategies on NSSI. Emotional dysregulation encompasses internal vulnerabilities related to various emotional aspects, including emotional perception, experience, and expression. Recent work suggested that the effects of emotion regulation strategies may emphasize the role of cognitive factors in modifying NSSI-related emotion dysregulation, indicating that these regulations are implemented at the cognitive level. One study reported that the experience of childhood maltreatment and stressful life events showed a significant indirect effect on NSSI through adaptive cognitive emotion regulation strategies (14). It was revealed that in the repetitive NSSI group, the effect of stress on NSSI frequency was mediated by emotion dysregulation, and the effect of stress on NSSI addictive features was mediated by both emotion dysregulation and maladaptive cognitive schemas in another research (15). However, it remains unclear whether these cognitive strategies moderate the mediating role of emotion dysregulation in the relationship between psychosocial factors and NSSI.

**Figure 1:**
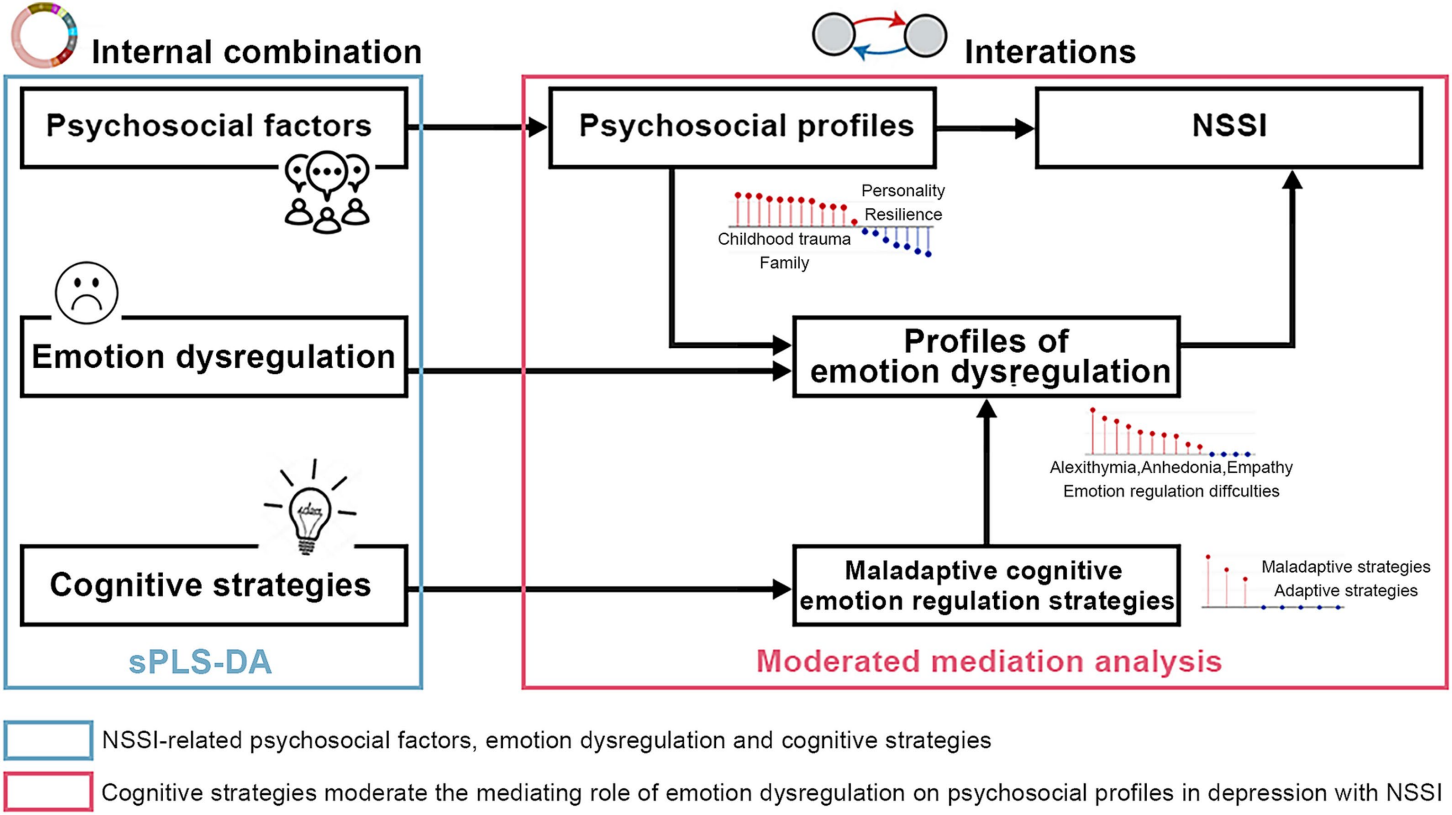
Pipeline for the study design. NSSI-related psychosocial factors, emotional impairments, and cognitive strategies were identified using sPLS-DA. A moderated mediation analysis was performed to investigate the interaction effect among psychosocial profiles, emotion dysregulation and cognitive strategies that are associated with NSSI. sPLS-DA, spare partial least squares discriminant analysis; NSSI, non-suicidal self-injury.

In the current study, we aimed to examine the correlation between psychosocial factors (individual and environmental), emotion dysregulation (intrapersonal and interpersonal), and cognitive regulation strategies (adaptive and maladaptive) in relation to NSSI using sparse partial least squares discriminant analysis (sPLS-DA). We then performed a moderated mediation analysis to explore how psychosocial factors and NSSI interact through emotion dysregulation, which is in turn moderated by cognitive regulation strategies (Figure 1).

## 2 Materials and methods

### 2.1 Participants

From October 2022 to June 2023, we recruited 122 outpatients or inpatients from Peking University Sixth Hospital diagnosed with depression, comprising 56 cases with NSSI (NSSI+) and 66 cases without NSSI (NSSI−). Depressive patients in partial remission could be included. They do not need to be higher than a cut-off. To meet the diagnostic criteria for NSSI behavior as recommended in the Diagnostic and Statistical Manual of Mental Disorders, Fifth Edition (DSM-5), NSSI+ participants had to have engaged in NSSI behaviors on more than 5 days within the past 12 months, whereas NSSI- participants had no history of self-injury. Additional inclusion criteria were: (a) aged 16–60 years; and (b) ICD-10 diagnosed with depressive episode or major depressive disorder according to the International Classification of Diseases, Tenth Edition (ICD-10). Exclusion criteria included: (a) patients who injure themselves in order to commit suicide; (b) accidental injuries that do not meet NSSI diagnostic criteria; (c) diagnosis of other current mental disorders, such as pervasive developmental disorders, psychotic disorders, manic episodes, substance dependence, or obsessive-compulsive disorders; and (d) presence of severe unstable physical illness. All participants provided written informed consent, and the study was approved by the Peking University Sixth Hospital Medical Ethics Committee.

### 2.2 Measures

**Figure 2:**
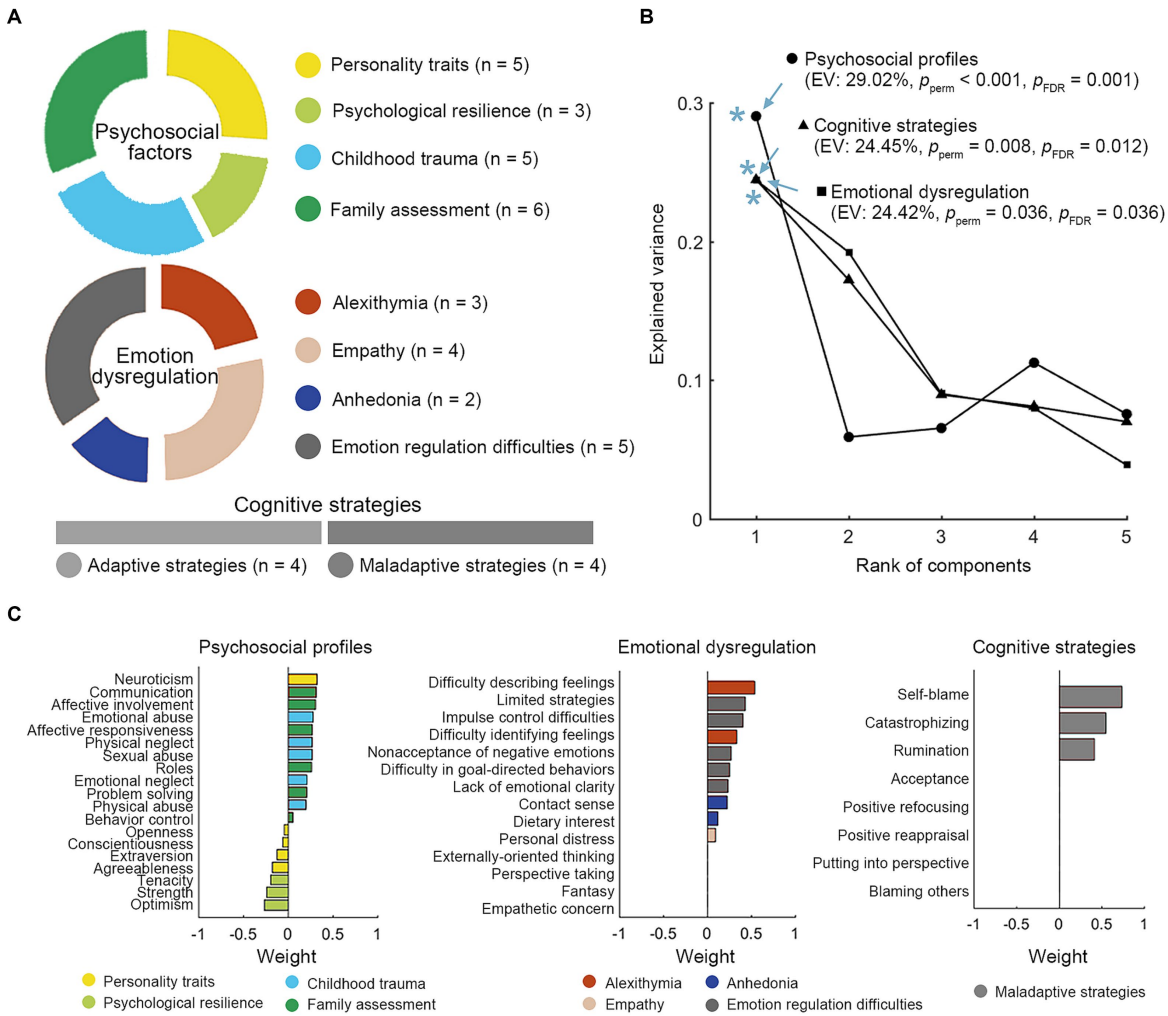
NSSI-related profiles of psychosocial factors, emotion dysregulation, and cognitive strategies. **(A)** Nineteen psychological factors belonging to 4 modules, fourteen items of emotional impairments from 4 categories and two kinds of cognitive regulations (each of which has 4 strategies) are included. **(B)** The sPLS-DA model identified a NSSI-related psychosocial profile (EV: 29.02%, *P*perm < 0.001, *P*_FDR_ = 0.001), profiles of emotional impairments (EV: 24.45%, *P*perm = 0.008, *P*_FDR_ = 0.012) and cognitive strategies (EV: 24.42%, *P*perm = 0.036, *P*_FDR_ = 0.036). The components significantly discriminate between the two groups of patients with and without NSSI. **(C)** The weight of significant items of each profile. * *p* < 0.05. EV, explained variance.

Depression and anxiety levels were assessed using the Zung self-rating anxiety scale (SAS) and the Zung self-rating depression scale (SDS) (16) and raw scores were employed in this study. The SAS has been shown to have good internal consistency with a Cronbach’s α of 0.82. The SDS scale was tested to show good reliability and validity, with a Cronbach’s α coefficient of 0.842 and a retest reliability correlation coefficient of 0.809 in the Chinese sample (17, 18). Psychosocial factors, emotion dysregulation, and cognitive regulation strategies were measured using nine self-assessment scales (Figure 2A).

#### 2.2.1 Psychosocial factors

Nineteen features from four questionnaires were integrated to assess psychosocial factors: (a) NEO Five Factor Inventory (NEOFFI) (19), assessing the five dimensions of personalities, including neuroticism, extraversion, openness, conscientiousness, and agreeableness. The NEOFFI is valid and reliable with excellent internal consistency scores of 0.82 to 0.89 in Chinese sample (20). (b) Connor-Davidson Resilience Scale (CD-RISC) (21) measuring the psychological resilience, including tenacity, strength, and optimism. The reliability coefficient of the Chinese version of CD-RISC was 0.91. The validity of CD-RISC was also satisfying in terms of the actual data. (c) Childhood Trauma Questionnaire (CTQ) (22), retrospectively assessing the childhood maltreatment, including emotional abuse, physical abuse, sexual abuse, emotional neglect, and physical neglect. The CTQ is valid and reliable with internal consistency scores of 0.64 in Chinese sample. (d) Family Assessment Device (FAD) (23), measuring the individual perceptions of his/her family functioning, comprising problem solving, communication, roles, affective responsiveness, affective involvement, behavioral control. The FAD has been shown to be valid and reliable.

#### 2.2.2 Emotion dysregulation

Emotion dysregulation was evaluated through four scales, including 14 features: (a) Toronto Alexithymia Scale (TAS) (24), assessing an individual’s ability to express and recognize internal and external emotions, including difficulty identifying feelings, difficulty describing feelings and externally-oriented thinking. The TAS attained good psychometric properties in the Chinese sample. (b) Interpersonal Reactivity Index-C (IRI-C) (25), evaluating the ability to empathize in four dimensions of perspective taking, fantasy, empathetic concern, and personal distress. The scale demonstrated satisfactory internal consistencies (ranging from 0.61 to 0.85). (c) Snaith-Hamilton Pleasure Scale (SHAPS) (26), measuring anhedonia, the inability to feel pleasure, categorized into, contact/sense and dietary/interest. The SHAPS also showed valid and reliable with an excellent internal consistency of 0.93. (d) The Difficulties in Emotion Regulation Scale (DERS-16) (27), covering lack of emotional clarity, difficulty in goal-directed behaviors, impulse control difficulties, limited strategies, and nonacceptance of negative emotions. The DERS-16 has retained excellent internal consistency, good test–retest reliability, and good convergent and discriminant validity in Chinese.

#### 2.2.3 Cognitive regulation strategies

Cognitive Emotion Regulation Questionnaire (CERQ) (28, 29) was used to assess eight strategies, including acceptance, positive refocusing, positive reappraisal, putting into perspective (adaptive), and rumination, self-blame, blaming others, catastrophizing (maladaptive). Higher scores indicated a higher likelihood of using a particular cognitive strategy to cope with negative events. The CERQ has been shown to be valid and reliable in both total scale and subscales.

### 2.3 Statistical analysis

SPSS 26.0 was used for descriptive statistics of participants’ demographic and all measures, presenting frequencies and proportions for categorical variables and means and standard deviations for normally distributed numerical variables. Independent samples *t*-test and chi-square test were employed to compare differences between NSSI+ and NSSI− groups in demographic and all scales.

Sparse partial least squares discriminant analysis (sPLS-DA) was conducted using the R package mixOmics to identify NSSI-related latent profiles of psychosocial factors, emotion dysregulation, and cognitive emotion regulation strategies. This method involved sparseness within the latent profiles and performed simultaneous dimension reduction to enable categorical classification with a specific focus on feature selection (30–34), and has been used successfully in the field of clinical assessment scales (35, 36), neuroimaging (35) and metabolomics (37), with the features range from 10 to 1,000. Here, the sPLS-DA identified components most associated with NSSI. The internal “loading” of each component measured how these features combine internally. Permutation tests (10,000 times) adjusted by false discovery rate (FDR) correction were used to test the statistical significance of the explained variance (EV) by the components.

A moderated mediation analysis was conducted to assess the complex associations of NSSI, psychosocial factors, emotion dysregulation and cognitive regulation strategies. R package Mediation (38) was used to test whether the association between psychosocial factors and NSSI (binary variable) can be mediated by emotion dysregulations and modulated by cognitive strategies. In the model, psychosocial factors were defined as an independent variable, the NSSI was defined as a dependent variable, the emotion dysregulation was defined as a mediator variable and the cognitive strategies were defined as a moderator variable. Patients’ age, sex, SAS, and SDS were controlled as covariates in our model. The method of Distribution of the Product was used to assess the coefficient of mediating effect, *ZaZb*, and its significance based on the distribution of the product with a 95% confidence interval (CI) that did not include zero. The moderated effect further determined whether cognitive strategies moderated the mediating path. Simple effect analysis calculated conditional indirect effects to test whether the indirect effect of emotion dysregulation disturbance varied under different levels of cognitive strategies. According to previous studies (39), two levels of cognitive strategies were defined, including low (one standard deviation below the mean) and high (one standard deviation above the mean) levels.

## 3 Results

### 3.1 Demographic and clinical characteristics

The study included 122 subjects with depression, with no statistically significant differences in age and gender between the groups. However, the levels of anxiety and depression were significantly higher in the NSSI+ group (*p* < 0.001). NSSI+ group exhibited more childhood trauma exposure (*p* = 0.002), worse family functioning (*p* = 0.006), higher neuroticism (*p* = 0.003) and worse psychological resilience (*p* = 0.021) than NSSI− participants. NSSI+ individuals demonstrated higher levels of alexithymia (*p* = 0.003) and anhedonia (*p* = 0.041), and more difficulties in emotion regulation (*p* < 0.001). Meanwhile, patients with NSSI tended to use maladaptive emotion regulation strategies (*p* = 0.002). The demographic and the characteristics of all measures in NSSI+ and NSSI− participants were presented in Table 1 and Supplementary Table S1.

**Table 1:** Demographic and clinical characteristics.

	Total (*N* = 122)	NSSI + (*N* = 56)	NSSI − (*N* = 66)	*t*/*χ^2^*	*p-*value
**Gender**				3.697	0.055
Male	27 (22.1%)	8 (29.6%)	19 (70.4%)		
Female	95 (77.9%)	48 (50.5%)	47 (49.5%)		
**Age**	26.0 ± 8.6	24.6 ± 7.7	27.2 ± 9.1	−1.686	0.094
**SAS**	45.4 ± 9.1	48.5 ± 9.3	42.8 ± 8.1	3.569	<0.001***
**SDS**	53.2 ± 10.6	56.8 ± 10.0	50.1 ± 10.1	3.697	<0.001***
**CTQ**	47.1 ± 14.0	51.3 ± 15.3	43.5 ± 11.6	3.125	0.002**
**FAD**	28.6 ± 7.2	30.5 ± 6.8	27.0 ± 7.3	2.803	0.006**
**NEOFFI**					
Neuroticism	32.9 ± 7.2	34.9 ± 6.9	31.1 ± 7.0	3.067	0.003**
Extraversion	17.0 ± 6.8	16.2 ± 7.2	17.7 ± 6.5	−1.190	0.236
Openness	27.4 ± 5.4	27.2 ± 5.3	27.6 ± 5.5	−0.409	0.683
Conscientiousness	35.1 ± 16.8	34.1 ± 18.1	35.9 ± 15.6	−0.591	0.556
Agreeableness	26.7 ± 5.0	25.8 ± 5.1	27.5 ± 4.8	−1.846	0.067
**CD-RISC**	42.6 ± 16.8	38.8 ± 18.2	45.8 ± 14.8	−2.346	0.021*
**TAS**	58.3 ± 11.7	61.6 ± 10.8	55.4 ± 11.8	2.990	0.003**
**IRI-C**	53.4 ± 12.2	53.4 ± 12.0	53.4 ± 12.4	0.028	0.978
**SHAPS**	30.2 ± 8.0	31.8 ± 8.1	28.8 ± 7.8	2.071	0.041*
**DERS**	54.6 ± 13.8	59.0 ± 13.2	50.9 ± 13.3	3.355	<0.001***
**CERQ**					
Adaptive strategies	51.1 ± 8.3	51.2 ± 8.5	51.1 ± 8.1	0.092	0.927
Maladaptive strategies	49.1 ± 10.2	52.2 ± 9.9	46.5 ± 9.8	3.164	0.002**

* *p* < 0.05, ** *p* < 0.01, *** *p* < 0.001; scale scores are mean ± standard deviation.SAS, self-rating anxiety scale; SDS, self-rating depression scale; CTQ, Childhood Trauma Questionnaire; FAD, Family Assessment Device; NEOFFI, NEO Five Factor Inventory; CD-RISC, Connor-Davidson Resilience Scale; TAS, Toronto Alexithymia Scale; IRI-C, Interpersonal Reactivity Index-C; SHAPS, Snaith-Hamilton Pleasure Scale; DERS, The Difficulties in Emotion Regulation Scale; CERQ, Cognitive Emotion Regulation Questionnaire.

### 3.2 NSSI-related profiles in psychosocial factors, emotion dysregulation, and cognitive regulation strategies

The NSSI-related psychosocial and emotion regulation profiles were identified through sPLS-DA (Figures 2B,C). An NSSI-related psychosocial profile identified through sPLS-DA was characterized by high neuroticism, more childhood trauma, poor family functioning, and low psychological resilience (EV: 29.02%, *P*_perm_ < 0.001, *P*_FDR_ = 0.001). Emotion dysregulation related with NSSI exhibited high levels of alexithymia, anhedonia, and emotional regulation difficulties (EV: 24.42%, *P*_perm_ = 0.036, *P*_FDR_ = 0.036). Maladaptive strategies of cognitive regulation, including self-blame, catastrophizing, and rumination, explained 24.42% variance of origin data structure (*P*_perm_ = 0.008, *P*_FDR_ = 0.012).

### 3.3 Moderated mediation model

**Figure 3:**
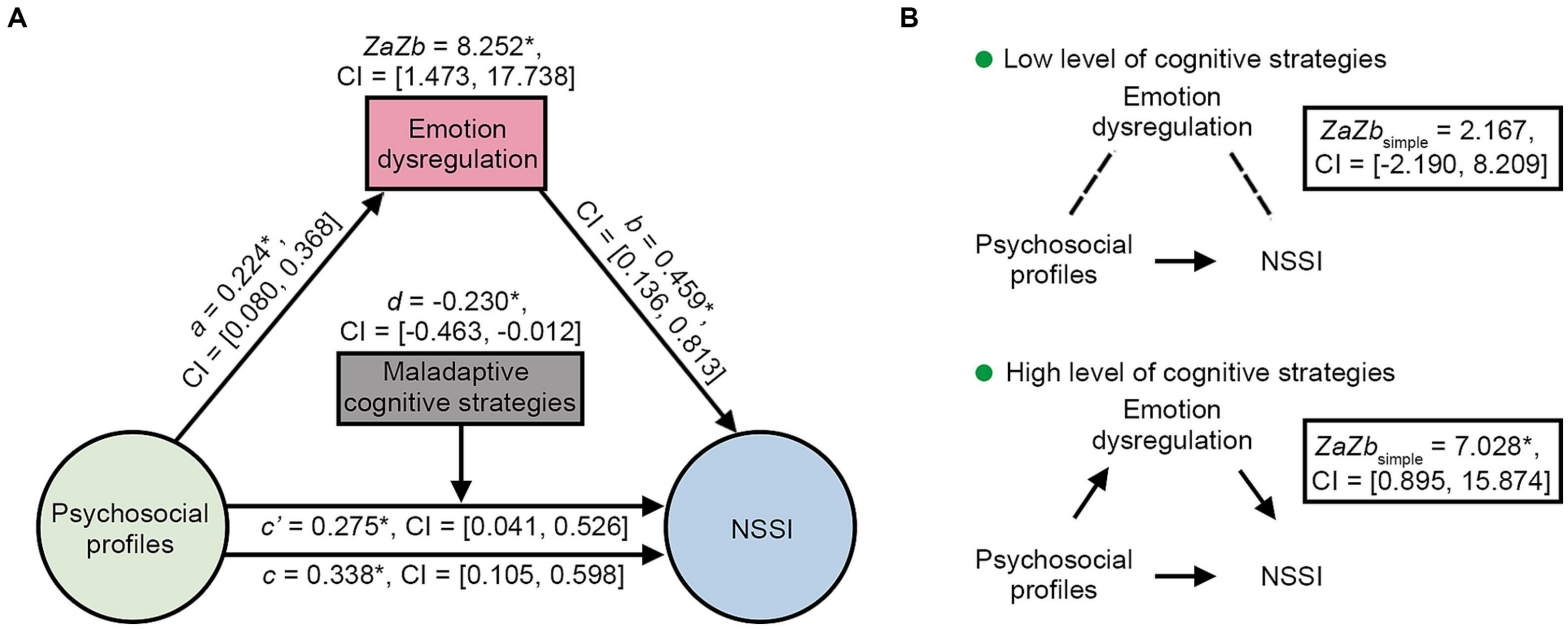
Moderated mediation model. **(A)** The emotional impairments were partially mediated the relationship between the psychosocial profiles and the NSSI, and the mediating effect was significantly moderated by cognitive strategies. **(B)** Simple effect analysis showed that the mediating effect of emotional impairments was significant when NSSI-related negative cognitive strategies is high. * *p* < 0.05. CI, confidence interval; NSSI, non-suicidal self-injury.

The moderated mediation analysis examined the interaction between psychosocial factors and NSSI through emotion dysregulation, moderated by cognitive regulation strategies (Figure 3A). The results revealed that the psychosocial profile was positively associated with the emotion dysregulation profile (*a* = 0.224, CI = [0.080,0.368]). The psychosocial profile was also positively associated with NSSI (*c* = 0.338, CI = [0.105,0.598]), as was the emotion dysregulation profile (*b* = 0.459, CI = [0.136,0.813]). The relationship between the psychosocial profile and NSSI was partially mediated by the emotion dysregulation profile (*ZaZb* = 8.252, CI = [1.473,17.738]). The mediated effect was moderated by maladaptive cognitive regulation strategies (*d* = −0.230, CI = [−0.463, −0.012]). As shown in Figure 3B, Simple effect analysis further revealed that the mediating effect of emotion dysregulation was significant under high levels of maladaptive cognitive strategies (*ZaZb*_simple_ = 7.028, CI = [0.895,15.874]), while it was not significant under low levels of maladaptive cognitive strategies (*ZaZb*_simple_ = 2.167, CI = [−2.190,8.209]).

## 4 Discussion

The present study explored internal combinations and external interactions within NSSI-related risk factors. The results identified a psychosocial profile characterized by high neuroticism, low psychological resilience, adverse childhood trauma and poor family communication. Additionally, an emotion dysregulation profile was associated with high levels of alexithymia, anhedonia, and emotional regulation difficulties. Emotion dysregulation partially mediated the association between the psychosocial profile and NSSI, and this mediating effect was moderated by maladaptive cognitive emotion regulation strategies. These findings provide a new perspective on understanding the composition and interactions of NSSI-related risk factors.

The observed psychosocial characteristics of heightened sensitivity and emotional instability, coupled with negative family environment and childhood maltreatment, are consistent with previous research. Neuroticism has been found to be higher in individuals with NSSI compared to those without NSSI (40). Individuals who scored high on neuroticism are more likely to experience anxiety, depression, and mood swings, and they may react strongly to stressful situations and have difficulty coping with challenges (41). Moreover, childhood maltreatment has been extensively studied as a risk factor for NSSI, with a meta-analysis indicating a strong association between childhood maltreatment and NSSI (42). Additionally, family factors, including family functioning and social support, have been linked to NSSI (43), with poor family functioning being associated with an increased risk of NSSI (11, 44–46). Considering the findings from our study and previous research, high neuroticism is associated with negative stress responses and difficulties in various life situations, and it is a possibility that these difficulties in managing emotions and stress can potentially lead to disharmonious relationships with family and their surroundings (47, 48). The present study adds to this understanding by examining the internal combinations of these risk factors related to NSSI.

In addition, we attempted to explore the internal features of emotion dysregulation by combining multiple risk factors, which may have a mediating effect between psychosocial profile and NSSI. Specifically, this internal combination presents a tendency toward worse ability to express and recognize internal and external emotions, increasing pleasure deficit, and more severe emotional regulation difficulties, which has been repeatedly emphasized in this study. A growing body of research also points to the association of different emotion disorders with NSSI. One study has been showed that depression plays a mediating role in the relationship between childhood maltreatment and NSSI, moderated by self-compassion (49). Another study drew a conclusion that emotional regulation mediated the association of borderline personality traits and NSSI in adolescents with depression (50). Borderline personality disorder (BPD) is frequently associated with NSSI and emotion dysregulation is also one of the core characteristics of BPD, which often contributes to self-injurious behaviors (51). These results implied that diverse emotion dysregulation may jointly influence the relationship between complex psychosocial factors and NSSI. Therefore, these findings are additionally illuminating for our understanding of the complex pattern of interactions within NSSI-related risk factors.

Further, we investigated the role of emotion dysregulation as a mediator between psychosocial factors and NSSI. Our results support the integrated theoretical model (7), wherein psychosocial factors contribute to emotion dysregulation, ultimately leading to NSSI (1, 8, 52). Furthermore, the moderating role of cognitive regulation strategies in this model is consistent with the affect regulation hypothesis (13). Maladaptive cognitive strategies in depression may modulate the mediating role of emotion regulation difficulties between psychosocial factors and NSSI. Depressed individuals often exhibit negative thinking styles and cognitive distortions (53), which may exacerbate their emotion regulation difficulties and further increase the risk of engaging in NSSI. Firstly, maladaptive cognitive strategies may exacerbate emotion dysregulation in individuals with depression. For example, individuals who engage in excessive rumination or negative self-talk may experience heightened negative emotions and have difficulty effectively regulating these emotions (54). This, in turn, may increase the likelihood of engaging in NSSI as a maladaptive coping mechanism (55). Secondly, maladaptive cognitive strategies can influence the interpretation and appraisal of psychosocial factors. Individuals with depression who employ cognitive distortions, such as overgeneralization, may perceive psychosocial stressors as more severe or personally threatening (56). This biased perception can further contribute to heightened emotional distress and increase the risk of NSSI. Moreover, maladaptive cognitive strategies may undermine the effectiveness of adaptive coping strategies and problem-solving skills. People who depend on avoiding or suppressing their emotions may find it challenging to effectively deal with psychosocial stressors or reach out to supportive sources for assistance (57). This can perpetuate a cycle of emotion dysregulation and increase the likelihood of engaging in NSSI as a maladaptive response. Therefore, a lower level of maladaptive cognitive strategies can buffer the effects of emotion dysregulation on NSSI development (58). Here, the involvement of adaptive cognitive strategies in this moderating process has not been demonstrated, possibly explained by their less frequent use in the current sample, which is consistent with findings from previous research in NSSI (59, 60) and depression (61, 62). However, it is important to note that the potential positive effects of adaptive strategies cannot be ruled out. Further research is needed to explore their role in influencing the relationship between psychosocial factors and NSSI. In summary, maladaptive cognitive strategies have the potential to moderate the mediating role of emotion dysregulation on the association between psychosocial factors and NSSI in depression. Understanding the interplay between these factors can provide valuable insights for the development of targeted interventions and treatment approaches that address cognitive processes, emotion regulation skills, and psychosocial stressors in individuals at risk for NSSI in the context of depression.

In addition to exploring the influencing factors of NSSI in depression (50), depression has also been proposed to mediate the relationship between the risk factors, such as childhood maltreatment, and NSSI (49). In this paper, although the levels of anxiety and depression were higher in NSSI+ individuals than NSSI− individuals in the current study, the depression symptoms appeared to be independent of the role of all the risk factors and modulators. These findings emphasize the importance of cognitive factors in patients with depression and provided deeper insights into the complex interactions between internal vulnerability factors and external risk factors that contribute to NSSI, as well as the relationship of symptoms of mood disorders and NSSI.

Several issues still need to be considered. Firstly, the sample size is relatively small so we cannot explore the potential effects of gender and age on NSSI (63, 64), and especially in the context of moderating effects, may result in relatively insufficient statistical power (65). Conducting studies with larger sample sizes in the future would be beneficial. Secondly, incorporating structured scales to assess NSSI frequency and severity would enhance the findings of the present study. Thirdly, since the co-occurrence of depressive disorders and BPD is a well-documented phenomenon (66), we cannot definitively rule out the possibility that some of the outpatient participants included in our study may indeed have concurrent BPD or other personality disorder diagnoses due to the time constraints inherent in outpatient visits.

In conclusion, this study identified internal combinations and external interactions within NSSI-related risk factors. The results found a psychosocial profile characterized with high neuroticism, childhood trauma, negative family environment and low psychological resilience, along with an emotional dysregulation profile consisting of high alexithymia, anhedonia, and emotional regulation difficulties. Emotion dysregulation partially mediated the relationship between this psychosocial profile and NSSI, and this mediating effect was moderated by maladaptive cognitive regulation strategies. Cognitive-behavioral therapy (CBT) (67) is a type of psychotherapy that can help individuals identify and replace maladaptive cognitive strategies with more adaptive ones. It has been shown to be effective in treating a variety of mental health conditions, including depression and anxiety. In addition to CBT, other techniques that can help improve maladaptive cognitive strategies include mindfulness-based interventions, Acceptance and Commitment Therapy (ACT), and dialectical behavior therapy (DBT). These techniques can help individuals become more aware of their thoughts and feelings, accept them without judgment, and learn how to respond to them in a more adaptive way. These findings have implications for the prevention, treatment, and rehabilitation of NSSI and may inspire future clinical interventions for NSSI in depression.

## Data availability statement

The raw data supporting the conclusions of this article will be made available by the authors, without undue reservation.

## Ethics statement

The studies involving humans were approved by Peking University Sixth Hospital Medical Ethics Committee. The studies were conducted in accordance with the local legislation and institutional requirements. The participants provided their written informed consent to participate in this study.

## Author contributions

YG: Conceptualization, Data curation, Formal analysis, Investigation, Methodology, Project administration, Supervision, Validation, Writing – original draft, Writing – review & editing. YX: Formal analysis, Methodology, Visualization, Writing – review & editing. ML: Writing – review & editing. LY: Writing – review & editing. PS: Writing – review & editing. XL: Resources, Writing – review & editing, Data curation. HY: Funding acquisition, Resources, Supervision, Writing – review & editing.
