# Supplementary material for: Maladaptive cognitive regulation moderates the mediating role of emotion dysregulation on the association between psychosocial factors and non-suicidal self-injury in depression
Source: Front Psychiatry. 2023 Nov 30;14:1279108. doi: 10.3389/fpsyt.2023.1279108 (PMC10719840; doi:10.3389/fpsyt.2023.1279108)
Supplement: Supplementary file 1 [file Table_1.DOCX]

Supplementary Material

# Supplementary Table 1

| **Supplement Table 1 Differences in the emotional dysregulation between groups** | | | | | |
| --- | --- | --- | --- | --- | --- |
|  | **Total**  **(N = 122)** | **NSSI+**  **(N = 56)** | **NSSI−**  **(N = 66)** | ***t*/*χ^2^*** | ***P* - value** |
| **CTQ** | 47.1 ± 14.0 | 51.3 ± 15.3 | 43.5 ± 11.6 | 3.125 | 0.002** |
| emotional abuse | 10.6 ± 4.6 | 11.8 ± 4.9 | 9.6 ± 4.2 | 2.669 | 0.009** |
| physical abuse | 7.4 ± 3.2 | 8.0 ± 3.8 | 6.9 ± 2.5 | 1.766 | 0.081 |
| sexual abuse | 6.5 ± 2.9 | 7.2 ± 3.7 | 5.9 ± 1.6 | 2.418 | 0.018* |
| emotional neglect | 14.1 ± 5.2 | 15.1 ± 5.0 | 13.2 ± 5.3 | 1.976 | 0.049* |
| physical neglect | 8.6 ± 3.1 | 9.4 ± 3.3 | 8.0 ± 2.8 | 2.579 | 0.011* |
| **NEO-FFI** |  |  |  |  |  |
| Neuroticism | 32.9 ± 7.2 | 34.9 ± 6.9 | 31.1 ± 7.0 | 3.067 | 0.003** |
| Extroversion | 17.0 ± 6.8 | 16.2 ± 7.2 | 17.7 ± 6.5 | **−**1.190 | 0.236 |
| Openness | 27.4 ± 5.4 | 27.2 ± 5.3 | 27.6 ± 5.5 | **−**0.409 | 0.683 |
| Agreeableness | 26.7 ± 5.0 | 25.8 ± 5.1 | 27.5 ± 4.8 | **−**1.846 | 0.067 |
| Conscientiousness | 35.1 ± 16.8 | 34.1 ± 18.1 | 35.9 ± 15.6 | **−**0.591 | 0.556 |
| **FAD** | 28.6 ± 7.2 | 30.5 ± 6.8 | 27.0 ± 7.3 | 2.803 | 0.006** |
| Problem Solving | 14.0 ± 2.9 | 14.6 ± 2.8 | 13.6 ± 2.9 | 1.907 | 0.059 |
| Communication | 23.5 ± 5.2 | 24.9 ± 4.2 | 22.2 ± 5.7 | 3.047 | 0.003** |
| Roles | 25.1 ± 4.4 | 26.2 ± 4.7 | 24.2 ± 3.8 | 2.459 | 0.015* |
| Affective Responsiveness | 16.3 ± 3.8 | 17.2 ± 3.5 | 15.5 ± 4.0 | 2.599 | 0.011* |
| Affective Involvement | 17.2 ± 3.7 | 18.2 ± 3.4 | 16.3 ± 3.7 | 2.949 | 0.004** |
| Behavioral Control | 17.6 ± 2.6 | 17.8 ± 3.0 | 17.5 ± 2.2 | 0.447 | 0.656 |
| **CD-RISC** | 42.6 ± 16.8 | 38.8 ± 18.2 | 45.8 ± 14.8 | **−**2.346 | 0.021* |
| Tenacity | 20.5 ± 9.7 | 18.4 ± 10.5 | 22.4 ± 8.7 | **−**2.294 | 0.024* |
| Strength | 15.0 ± 5.3 | 14.1 ± 5.8 | 15.7 ± 4.7 | **−**1.707 | 0.090 |
| Optimism | 7.1 ± 3.1 | 6.3 ± 3.3 | 7.7 ± 2.7 | **−**2.571 | 0.011* |
| **TAS** | 58.3 ± 11.7 | 61.6 ± 10.8 | 55.4 ± 11.8 | 2.990 | 0.003** |
| Difficulty identifying feelings | 21.1 ± 5.7 | 22.7 ± 5.3 | 19.8 ± 5.8 | 2.795 | 0.006** |
| Difficulty describing feelings | 15.4 ± 4.1 | 16.9 ± 3.9 | 14.1 ± 3.9 | 3.908 | 0.000*** |
| Externally-oriented thinking | 21.2 ± 3.9 | 21.6 ± 3.8 | 20.8 ± 4.0 | 1.101 | 0.273 |
| **IRI-C** | 53.4 ±12.2 | 53.4 ± 12.0 | 53.4 ± 12.4 | 0.028 | 0.978 |
| Perspective Taking | 10.9 ± 4.3 | 10.4 ±4.2 | 11.3 ± 4.4 | -1.091 | 0.277 |
| Fantasy | 14.4 ± 4.8 | 14.5 ± 4.9 | 14.4 ± 4.7 | 0.092 | 0.927 |
| Empathetic Concern | 16.0 ± 4.8 | 15.7 ± 4.7 | 16.1 ± 5.0 | **−**0.461 | 0.646 |
| Personal Distress | 12.2 ± 4.4 | 12.9 ± 4.3 | 11.6 ± 4.5 | 1.562 | 0.121 |
| **SHAPS** | 30.2 ± 8.0 | 31.8 ± 8.1 | 28.8 ± 7.8 | 2.071 | 0.041* |
| contact/sense | 13.0 ± 3.9 | 13.8 ± 4.0 | 12.2 ± 3.7 | 2.207 | 0.029* |
| dietary/interest | 17.2 ± 4.7 | 18.0 ± 4.9 | 16.6 ± 4.5 | 1.693 | 0.093 |
| **DERS** | 54.6 ± 13.8 | 59.0 ± 13.2 | 50.9 ± 13.3 | 3.355 | 0.001*** |
| Lack of emotional clarity | 5.8 ± 2.3 | 6.3 ± 2.5 | 5.3 ± 2.1 | 2.274 | 0.025* |
| Difficulty in goal-directed behaviors | 11.6 ± 2.8 | 12.2 ± 2.7 | 11.0 ± 2.8 | 2.353 | 0.020* |
| Impulse control difficulties | 10.3 ± 3.2 | 11.3 ± 3.0 | 9.5 ± 3.2 | 3.146 | 0.002** |
| Limited strategies | 18.2 ± 4.9 | 19.7 ± 4.6 | 16.9 ± 4.8 | 3.333 | 0.001*** |
| Nonacceptance of negative emotions | 8.8 ± 3.0 | 9.5 ± 3.1 | 8.2 ± 2.8 | 2.448 | 0.016* |
| **CERQ** |  |  |  |  |  |
| Self-Blame | 14.2 ± 3.2 | 15.1 ± 3.0 | 13.3 ± 3.1 | 3.210 | 0.002** |
| Acceptance | 14.5 ± 3.0 | 14.7 ± 3.3 | 14.3 ± 2.8 | 0.800 | 0.425 |
| Rumination | 14.0 ± 4.1 | 14.9 ± 4.1 | 13.3 ± 4.0 | 2.142 | 0.034* |
| Positive Refocusing | 12.0 ± 3.6 | 11.8 ± 3.7 | 12.1 ± 3.5 | **−**0.365 | 0.716 |
| Positive Reappraisal | 12.4 ± 3.7 | 12.2 ± 3.8 | 12.6 ± 3.7 | **−**0.577 | 0.565 |
| Putting into perspective | 12.3 ± 3.0 | 12.5 ± 2.9 | 12.6 ± 3.1 | 0.590 | 0.556 |
| Catastrophizing | 10.9 ± 3.8 | 11.8 ± 3.7 | 10.1 ± 3.7 | 2.580 | 0.011* |
| Blaming Others | 10.0 ± 3.6 | 10.3 ± 3.7 | 9.8 ± 3.5 | 0.839 | 0.403 |
| Adaptive strategies | 65.6 ± 10.5 | 66.4 ± 10.7 | 64.8 ± 10.3 | 0.863 | 0.390 |
| Maladaptive strategies | 49.1 ± 10.2 | 52.2 ± 9.9 | 46.5 ± 9.8 | 3.164 | 0.002** |
| *：*p*<0.05，**：*p*<0.01，***：*p*<0.001；scale scores are mean ± standard deviation. SAS: self-rating anxiety scale; SDS: self-rating depression scale; CTQ: Childhood Trauma Questionnaire; FAD: Family Assessment Device; NEOFFI: NEO Five Factor Inventory; CD-RISC: Connor-Davidson Resilience Scale; TAS: Toronto Alexithymia Scale; IRI-C: Interpersonal Reactivity Index-C; SHAPS: Snaith-Hamilton Pleasure Scale; DERS: The Difficulties in Emotion Regulation Scale; CERQ: Cognitive Emotion Regulation Questionnaire. | | | | | |
